# On Cod-liver Oil in Phthisis

**Published:** 1849-07

**Authors:** C. J. B. Williams


*On Cod Liver Oil in Phthisis.*—By Dr. C. J. B. Williams.—Dr. Williams has retained notes of 234 cases of phthisis in which he has given cod liver oil. In 9 cases the oil disagreed, and was discontinued ; in 19, although taken, it appeared to do no good, whilst in 206, out of 234, its use was followed by marked improvement, varying in degree from temporary retardation of the progress of the disease, and a mitigation of distressing symptoms, to a more or less complete restoration to apparent health.

The most numerous examples of decided and lasting improvement occurred in patients in the second stage of the disease. Even in a few days the cough was mitigated, the expectoration diminished in quantity and opacity, the night sweats ceased, the pulse became slower and of better volume, and the appetite, flesh and strength, were gradually improved. There was generally a diminution and gradual cessation of the crepitus; the breath-sound becoming drier and clearer; but the dullness and tubular character of the breath and voice sounds seldom exhibited any marked decrease till after several weeks' use of the oil, in conjunction with counter-irritation. In several cases in which the disease has existed long, the restoration has never been perfect; even when all the common symptoms have entirely disappeared, there have remained perceptible inequalities in the breath and stroke sounds generally with prolonged expiratory sound, which is more or less tubular towards the root of the lung. "These signs," says Dr. Williams, "I have learned, by the experience of many years, not to consider as exceptional against recovery, for they appear to be dependent on the puckering of the texture, often with pleural adhesions and old deposits in the bronchial glands, so frequently found after death at the summits and near the roots of the lungs of persons who have not for many years exhibited symptoms of any pectoral disease."

In the first stage of the disease, the results seem equally satisfactory. The good effect of the oil is commonly manifested in the abatement of cough and feverish excitement, and in the improvement of flesh and strength.—The physical signs of improvement are the same as those which take place tardily in the second stage, after the removal of the hard ronchi; and Dr. Williams believes that the treatment by the oil, combined with counter-irritation, seems to bring back the lungs from the stage of incipient softening to that of simple deposit, which is tardier in its changes of increase or diminution, and may remain long stationary without any obvious alteration.

In the third stage, the effects of the oil are often very marvellous. The whole number of cases with one or more cavities, as indicated by physical signs, which, under Dr. Williams' hands, manifestly improved, amounts to sixty-two. In thirty-four of these there was continued improvement up to the last reports. Eleven cases, which exhibited decided improvement for a time, have since again declined or terminated in death. Of the remaining seventeen, he has had no recent report, and does not know whether the amelioration has been permanent or not.—*Monthly Journal.—Western Lancet.*

